# Monitoring Silent Spillovers Before Emergence: A Pilot Study at the Tick/Human Interface in Thailand

**DOI:** 10.3389/fmicb.2019.02315

**Published:** 2019-10-17

**Authors:** Sarah Temmam, Delphine Chrétien, Thomas Bigot, Evelyne Dufour, Stéphane Petres, Marc Desquesnes, Elodie Devillers, Marine Dumarest, Léna Yousfi, Sathaporn Jittapalapong, Anamika Karnchanabanthoeng, Kittipong Chaisiri, Léa Gagnieur, Jean-François Cosson, Muriel Vayssier-Taussat, Serge Morand, Sara Moutailler, Marc Eloit

**Affiliations:** ^1^Institut Pasteur, Biology of Infection Unit, Inserm U1117, Pathogen Discovery Laboratory, Paris, France; ^2^Institut Pasteur – Bioinformatics and Biostatistics Hub – Computational Biology Department, Institut Pasteur, USR 3756 CNRS, Paris, France; ^3^Institut Pasteur, Production and Purification of Recombinant Proteins Technological Platform – C2RT, Paris, France; ^4^Centre de Coopération Internationale en Recherche Agronomique pour le Développement (CIRAD), UMR InterTryp, Bangkok, Thailand; ^5^InterTryp, Institut de Recherche pour le Développement (IRD), CIRAD, University of Montpellier, Montpellier, France; ^6^Department of Parasitology, Faculty of Veterinary Medicine, Kasetsart University, Bangkok, Thailand; ^7^UMR BIPAR, Animal Health Laboratory, ANSES, INRA, Ecole Nationale Vétérinaire d'Alfort, Université Paris-Est, Maisons-Alfort, France; ^8^Faculty of Veterinary Medicine, Kasetsart University, Bangkok, Thailand; ^9^Faculty of Tropical Medicine, Mahidol University, Bangkok, Thailand; ^10^Institut des Sciences de l'Evolution, CNRS, CC065, Université Montpellier, Montpellier, France; ^11^CIRAD ASTRE, Faculty of Veterinary Technology, Kasetsart University, Bangkok, Thailand; ^12^National Veterinary School of Alfort, Paris-Est University, Maisons-Alfort, France

**Keywords:** virome, tick, emergence, spillover, LIPS

## Abstract

Emerging zoonoses caused by previously unknown agents are one of the most important challenges for human health because of their inherent inability to be predictable, conversely to emergences caused by previously known agents that could be targeted by routine surveillance programs. Emerging zoonotic infections either originate from increasing contacts between wildlife and human populations, or from the geographical expansion of hematophagous arthropods that act as vectors, this latter being more capable to impact large-scale human populations. While characterizing the viral communities from candidate vectors in high-risk geographical areas is a necessary initial step, the need to identify which viruses are able to spill over and those restricted to their hosts has recently emerged. We hypothesized that currently unknown tick-borne arboviruses could silently circulate in specific biotopes where mammals are highly exposed to tick bites, and implemented a strategy that combined high-throughput sequencing with broad-range serological techniques to both identify novel arboviruses and tick-specific viruses in a ticks/mammals interface in Thailand. The virome of Thai ticks belonging to the *Rhipicephalus, Amblyomma, Dermacentor, Hyalomma*, and *Haemaphysalis* genera identified numerous viruses, among which several viruses could be candidates for future emergence as regards to their phylogenetic relatedness with known tick-borne arboviruses. Luciferase immunoprecipitation system targeting external viral proteins of viruses identified among the *Orthomyxoviridae, Phenuiviridae, Flaviviridae, Rhabdoviridae*, and *Chuviridae* families was used to screen human and cattle Thai populations highly exposed to tick bites. Although no positive serum was detected for any of the six viruses selected, suggesting that these viruses are not infecting these vertebrates, or at very low prevalence (upper estimate 0.017% and 0.047% in humans and cattle, respectively), the virome of Thai ticks presents an extremely rich viral diversity, among which novel tick-borne arboviruses are probably hidden and could pose a public health concern if they emerge. The strategy developed in this pilot study, starting from the inventory of viral communities of hematophagous arthropods to end by the identification of viruses able (or likely unable) to infect vertebrates, is the first step in the prediction of putative new emergences and could easily be transposed to other reservoirs/vectors/susceptible hosts interfaces.

## Introduction

Among the list of human-infecting pathogens, two-thirds are zoonotic agents (Woolhouse et al., 2012). With increasing contacts between humans, wildlife, and their associated arthropods (due to changes in land use, global climate warming, or urbanization), the frequency of human infections with animal viruses is expected to increase (Cutler et al., 2010). Wolfe et al. noted that the emergence of a pathogen of vertebrate into a new susceptible vertebrate population goes through four main steps: (1) spillover of a pathogen from its animal reservoir to sporadic human cases, (2) limited interhuman transmission of a zoonotic pathogen, (3) large-scale spread of a zoonotic pathogen through interhuman transmissions, and (4) adaptation of the pathogen to humans (Wolfe et al., 2007). This is not the case for arboviruses: arbovirus emergence is linked to the spread of infected arthropods (directly or via the geographical expansion of their vertebrate host, as for ticks) into new areas or to the incursion of humans in new biotopes. Most recent epidemics that emerged were linked to arboviruses that were already known but described in limited areas, as the recent example of Zika virus rapid expansion (Liu et al., 2019).

Ticks are major hematophagous arthropod vectors that harbor multiple infectious agents. Some of these agents are known to infect humans, leading to several tick-borne diseases such as Lyme borreliosis (*Borrelia burgdorferi sensu lato*), tick-borne encephalitis (due to several flaviviruses such as tick-borne encephalitis virus or Powassan virus), and spotted fever (several *Rickettsia*) (Moyer, 2015; Nelder et al., 2016). However, asymptomatic or pauci-symptomatic infections with non-specific syndromes (for example, asthenia, fever, myalgia, etc.) occurring after tick bites are frequent and have become a serious issue (Moyer, 2015). The same situation is observed for domestic animals: since several tick species, mainly ixodid ticks, are able to feed both on domestic animals and humans (Kiewra and Lonc, 2012), they can transmit zoonotic pathogens common to animals and humans. Moreover, due to global and local environmental changes, the geographical repartition of ticks is increasing, leading to exposure of naive populations to new pathogens (Ogden et al., 2013). The progresses in usage of high-throughput sequencing (HTS) have allowed an increase in the knowledge of infectious agents, and particularly viruses, carried by ticks. Recent studies have indeed reported the identification of new tick-borne viruses, belonging either to known or completely new viral families, for which the potential zoonotic risk for humans or domestic animals is still unknown (Tokarz et al., 2014a, 2018; Xia et al., 2015; Moutailler et al., 2016; Shi et al., 2016; Pettersson et al., 2017). For example, among the *Phenuiviridae*, known to contain tick-borne pathogens such as the severe fever with thrombocytopenia syndrome phlebovirus (SFTS virus) (Silvas and Aguilar, 2017), recent bisegmented phleboviruses have been described, such as Lihan tick phlebovirus (LTPV) primarily identified in *Rhipicephalus microplus* ticks from China, Brazil, and Trinidad and Tobago (Li C. X. et al., 2015; Souza et al., 2018; Sameroff et al., 2019) and further detected in Turkish *Hyalomma marginatum* (Dinçer et al., 2017) and *Rhipicephalus sanguineus* ticks (Brinkmann et al., 2018). *Flaviviridae*-related tick-borne viruses (e.g., Bole tick virus 4, BLTV4) were primarily associated with *Hyalomma asiaticum* and *Rh. sanguineus* ticks (Shi et al., 2015; Sameroff et al., 2019). This virus presents a genome 1.5 times larger than other *Flaviviridae* tick-borne viruses and could constitute, with other related flaviviruses that present large genomes, at least a new genus among the *Flaviviridae* family. In complement to known rhabdoviruses transmitted by ticks (Labuda and Nuttall, 2004) [including several viruses pathogenic for humans (Menghani et al., 2012)], novel single-stranded RNA (ssRNA) negative-strand viruses belonging to the dimarhabdovirus group within the *Rhabdoviridae* family were also identified in *Rhipicephalus* [*Rh. microplus* (Li C. X. et al., 2015), *Rh. annulatus* (Li C. X. et al., 2015; Brinkmann et al., 2018)] ticks [for example, Wuhan tick virus 1 (WhTV-1)]. In addition to these viral families known to contain tick-borne viruses, new viruses identified by HTS and constituting novel viral families recently recognized by the ICTV were reported. It is the case of the *Chuviridae* family, a group of viruses belonging to the *Jingchuvirales* order [class *Monjiviricetes*, phylum *Negarnaviricota*, (Siddell et al., 2019)] and constituted of circular or segmented ssRNA negative-strand viruses primarily associated with *Dermacentor* sp., *Haemaphysalis parva* (Li C. X. et al., 2015; Brinkmann et al., 2018), and *Rh. sanguineus* (Sameroff et al., 2019) ticks [e.g., Changping tick virus 2 (CpTV2)] or *Rh. microplus* ticks from China, Brazil, and Trinidad and Tobago [Wuhan tick virus 2 (WhTV2)] (Li C. X. et al., 2015; Souza et al., 2018; Sameroff et al., 2019).

We hypothesized that currently unknown tick-borne arboviruses could silently circulate in specific biotopes where mammals (including humans) are highly exposed to tick bites and used wide range identification techniques to track them in a tick/mammal interface in Thailand. Despite the fact that the description of the virome of ticks is a prerequisite to the evaluation of the risk of spillover, few studies have tried to go further and characterize, among the viral communities infecting ticks, which viruses would more likely be transmissible to vertebrates. Starting from the inventory of viruses infecting tick vectors, the first step in the understanding of the mechanisms of viral emergence is therefore to identify which viruses can cross the species barrier and infect vertebrates, even without any reported clinical signs. Serological techniques are useful tools for getting insights into arbovirus exposure history of new hosts without the limits of genomic tests, which need to collect biological samples in the course of a viremic phase. However, the recognition of the antigen (Ag) by its specific antibodies (Ab) requires good conservation of epitopes conformation, a problem frequently encountered in solid phase Ab/Ag assays (Burbelo et al., 2010). In 2010, Burbelo et al. have established a sensitive liquid phase format assay for profiling Ab responses to various antigens (Burbelo et al., 2010). Luciferase immunoprecipitation system (LIPS) assays use as antigens viral open reading frames (ORFs) fused to the luciferase (Luc) reporter gene expressed in mammalian cells. Fusion proteins are harvested as cell lysates under native conditions and used as antigens. Mixed with serum samples, the resulting immune complexes are then immunoprecipitated using protein A/G-coated beads and revealed by the substrate of the Luc. The measure of Luc activity is an index of the initial Ab titer. Owing to high affinity to the Fc region of their immunoglobulins to staphylococcal A and streptococcal G proteins, LIPS assay can be performed either on human and several animals sera (such as rabbits, dogs, monkeys, cows, mice, etc.) (Burbelo et al., 2010), resulting in the possibility to use one single tool to monitor the viral cycle both in its reservoir (frequently animal) and in its spillover (human) host.

In Thailand, several infectious agents such as protozoa (i.e., *Babesia* and *Theileria*) and bacteria (*Anaplasma, Ehrlichia, Rickettsia*, and *Bartonella*, for example) are carried by ticks. Viruses belonging to the *Reoviridae, Togaviridae, Flaviviridae, Nairoviridae*, and *Orthomyxoviridae* were also reported (Cornet et al., 2004; Ahantarig et al., 2008). We hypothesized that other tick-borne viruses (new or already known) are silently crossing the barrier and sporadically infect humans and/or domestic animals living in the vicinity of humans (as cattle or dogs for example) in specific ecosystems where populations are in close contact with ticks. The aim of this study is therefore to evaluate the ability of the strategy that consists in coupling HTS with serological screenings to identify which viruses are (and are not) able to cross the species barrier and infect new vertebrate hosts.

## Materials and Methods

### Sample Collection and Processing

#### Ticks Samples

A total of 266 ticks (18 males, 197 females, and 51 nymphs) were collected in Ban Huay Muang village (Tha Wang Pha district, Nan province, Thailand) in November 2012. Ticks were morphologically identified at least at the genus level: 40 *Rh. sanguineus*, 22 *Amblyomma* sp., 148 *Boophilus* sp., 16 *Dermacentor marginatus*, 22 *Hyalomma* sp., and 18 *Haemaphysalis* sp. To determine the species of *Amblyomma* sp., *Boophilus* sp., *Hyalomma* sp., and *Haemaphysalis* sp. ticks, all trimmed reads were mapped onto the *Ixodidae* Barcode of Life Data Systems (BOLD) database, *de novo* assembled after extraction of mapped reads, and submitted to the BOLD Identification System. *Boophilus* sp. ticks were identified as *Rh. microplus*, and *Haemaphysalis* sp. were identified as *Haemaphysalis hystricis*.

All of the genera collected here contain several tick species that can either bite wildlife or domestic animals (including cattle) and even humans (Kiewra and Lonc, 2012). Most of the ticks were engorged (222 out of 266) and were collected as follows: *Amblyomma* ticks were collected on wild boars and rodents; *Rh. microplus* ticks were collected on cattle (except for two ticks collected by flagging); *D. marginatus* ticks were collected on wild boars (except for one tick collected by flagging); *Ha. hystricis* ticks were collected either on wild boars (*N* = 11), on dogs (*N* = 2), and by flagging (*N* = 3); *Hyalomma* ticks were either collected on dogs (*N* = 3) or on wild boars (*N* = 15); and *Rh. sanguineus* ticks were either collected on dogs (*N* = 12) or on the floor of houses were dogs lived (*N* = 4).

All ticks were washed as previously described (Vayssier-Taussat et al., 2013) to remove external contaminants and homogenized individually in Dulbecco's modified Eagle's medium (DMEM) supplemented with 10% of fetal calf serum. After clarification, DNA and RNA extractions were performed from 100 μl of supernatant using the Nucleospin Tissue or the Nucleospin RNA II kits, respectively (Macherey-Nagel, Germany), according to the manufacturer's recommendations. Purified DNA and RNA were eluted into 50 μl of elution buffer and RNAse-free water, respectively. Extracts were kept at −80°C before HTS libraries preparation and further amplifications.

#### Human Serum Samples

The study procedures concerning human sample collection, laboratory investigation, interviews, and questionnaires were approved by the Ethical Committee of the Faculty of Tropical Medicine, Mahidol University (Bangkok, Thailand), document no 0517.1116/661.

Public health personnel made appointment with the villager participants in Saen Thong subdistrict, Nan province. Voluntary participation in the research project was obtained after explanation of the purposes of the study and by signing the informed consent/assent form. Blood samples were collected once by local nurses from a primary care unit, as previously described (Chaisiri et al., 2018).

#### Cattle Serum Samples

With the support and under the control of the local veterinary services from the Department of Livestock Development, 70 cattle were blood sampled in the Tha Wang Pha district, Nan province, in November 2012. When animals were infested, ticks were collected. Animals were tested by ELISA against *Trypanosoma evansi* as previously described (Desquesnes et al., 2009), and seropositive samples were tested by PCR using *Trypanosoma brucei* primers previously published (Masiga et al., 1992; Pruvot et al., 2010, 2013) to confirm active infection.

### High-Throughput Sequencing and Bioinformatics Analyses of Tick Virome

Individual RNA tick extracts were combined to form one pool of total RNA that was used as template for random reverse transcription followed by random amplification using Qiagen QuantiTect Whole Transcriptome kit. Complementary DNA (cDNA) was used for library preparations and sequenced on an Illumina HiSeq2000 sequencer in a single-read 100-bp format outsourced to DNAVision Company.

A total of 222,152,554 of raw reads were processed with an *in-house* bioinformatics pipeline comprising quality check and trimming [based on AlienTrimmer package (Criscuolo and Brisse, 2014)], reads normalization [using BBnorm program (https://jgi.doe.gov/data-and-tools/bbtools)], *de novo* assembly (using Megahit tool Li D. et al., 2015), and ORF prediction of contigs and singletons (https://figshare.com/articles/translateReads_py/7588592). A BLAST-based similarity search was performed for all contigs and singletons against the protein Reference Viral database (Bigot et al., 2019) followed by a BlastP-based verification of the accuracy of the viral taxonomic assignation against the whole protein NCBI/nr database. A final BLAST-based verification was performed against NCBI/nt to confirm that no better hit was obtained in non-coding regions of NCBI/nt.

The quantification of abundance of each viral taxon was obtained by summing the length (in amino acids) of all sequences being associated to this taxon instead of summing the raw number of sequences, to take into account the identification of long viral contigs. In addition, to take into account that contigs are the results of the assembly of numerous reads, we weighted the identification of viral hits coming from contigs by multiplying the length (in amino acids) of contigs with their respective *k*-mer coverage.

Phylogenetic reconstructions were performed on conserved non-structural RNA-dependent RNA polymerase (RdRP) gene. Complete ORFs were aligned along with other representative sequences of viral orders/families using MAFFT (Multiple Alignment using Fast Fourier Transform) aligner under the L-INS-i or G-INS-i parameters (Katoh et al., 2017). The best amino acids substitution models that fitted the data were determined with ATGC Smart Model Selection (Lefort et al., 2017) implemented in http://www.atgc-montpellier.fr/phyml-sms/ using the corrected Akaike information criterion. Phylogenetic trees were constructed using maximum likelihood method implemented through RAxML program under the CIPRES Science Gateway portal (Miller et al., 2010) according to the selected substitution model. Nodal support was evaluated using the “automatic bootstrap replicates” parameter. For *Orthomyxoviridae*-related phylogenetic analyses, neighbor-joining reconstruction method, *p*-distance, and 10,000 bootstraps replications were used.

### Genome Finishing and Prevalence Study in Ticks

The complete ORFs were obtained by conventional PCR and Sanger sequencing after designing specific primers targeting the identified viruses. Briefly, viral RNA was reverse transcribed using the SuperScript IV reverse transcriptase (Invitrogen, USA), and cDNA was subsequently used to fill the gaps in the genomes using the Phusion High Fidelity DNA polymerase (New England Biolabs, France). Positive PCR products were further purified and sequenced by Sanger sequencing on the Eurofins Segenic Cochin platform. When start and stop codons were lacking, rapid amplification of cDNA ends PCR were performed using the 5′/3′ RACE kit, second generation (Roche Applied Science, Germany).

For prevalence study, individual RNA extracts were reverse transcribed into cDNA using the qScript cDNA Supermix kit according to the manufacturer's instructions (Quanta Biosciences, USA). Briefly, the reaction was performed in a final volume of 5 μl containing 1 μl of qScript cDNA Supermix 5×, 1 μl of RNA, and 3 μl of RNase-free water. Cycling condition was as follows: one cycle at 25°C for 5 min, one cycle at 42°C for 30 min, and one final cycle at 85°C for 5 min. Specific Taqman qPCR systems targeting the polymerase gene were designed for six relevant viruses (the sequences of primers and probes are available upon request). Real-time Taqman qPCR assays were performed in a final volume of 12 μl using the LightCycler® 480 Probe Mastermix (Roche Applied Science) at 1× final concentration, with primers and probes at 200 nM, and 2 μl of cDNA. Negative (water) control was included in each run. Thermal cycling conditions were as follows: 95°C for 5 min, 45 cycles at 95°C for 10 s, then 60°C for 15 s, with a final cooling step at 40°C for 10 s.

To identify possible endogenous viral elements (EVEs) originating from arthropod hosts that could have been sequenced during the process, nested qPCRs were performed on tick-borne total DNA without any RT step targeting the polymerase gene of the six selected as relevant viruses. Positive or suspicious results were further analyzed by Sanger sequencing.

### Serological Screening of Human and Cattle Sera

#### Choice of Target Antigens

To maximize the probability of detecting antibodies specific to the viruses detected in ticks and minimize the risk of cross-reactions, we decided to target viral external proteins, meaning glycoproteins (GP) or nucleoproteins (NP) when GP were not identified. The annotation of the genes and the consecutive identification of surface proteins were first performed by position-specific iterated Blast (Psi-Blast). For viral ORFs for which we could not determine the function of the gene by Psi-Blast, Swiss Model was used to model the structure of the viral protein and to compare it to known structures deposited onto the PDB database (https://swissmodel.expasy.org/interactive) (Arnold et al., 2006; Biasini et al., 2014). Finally, for hypothetical proteins for which neither Psi-Blast nor Swiss Model gave positive results, online prediction of possible N- and O-glycosylation sites was performed both on the NetNGlyc and NetOGlyc webservers (http://www.cbs.dtu.dk/services/NetNGlyc/ or http://www.cbs.dtu.dk/services/NetOGlyc/) (Gupta et al., 2002).

The identified GP or NP were then analyzed with the Transmembrane Prediction tool of CLC Main Workbench 7 (Qiagen Bioinformatics) to identify extracellular regions of the proteins. These regions were used to produce viral antigens. The coordinates of the extracellular regions expressed for relevant viruses are presented in Supplemental Table 1.

#### Cloning

Complete extracellular regions of viral glycoproteins and/or nucleoproteins were cloned into pFC32K vector (Promega, France), at the N-terminal end of the NanoLuc luciferase gene, using the Gibson Assembly (GA) kit (NEBuilder® HiFi DNA Assembly Master Mix, New England Biolabs), according to the manufacturer's instructions. First, the Kosak sequence (GCCACC, in bold in Supplemental Table 2) and the start codon (ATG, in italic in Supplemental Table 2) were added by PCR in 5′ of the target fragment along with a 28- and a 27-nt vector region overlapping the sequence of the vector and containing the restrictions sites of the multiple cloning sites (underlined in Supplemental Table 2, SgfI and EcoICRI, respectively, for forward and reverse primers). Great attention was paid to maintain the reading frame between the target fragment and the NanoLuc gene. Then, PCR products were gel purified with the Nucleospin Gel and PCR cleanup kit (Macherey-Nagel) and quantified by Qubit with the ds DNA High Sensitivity kit (Invitrogen).

One hundred nanograms of pre-linearized pFC32K vector (digested by SgfI and EcoICRI restriction enzymes, Carboxy Flexi Enzyme Blend, Promega) was used to clone by GA the target fragments at a ratio of 1:3 to 1:10 (depending on the constructs) in XL1 competent cells (Agilent Technologies, USA). Positive clones were screened by PCR with primers designed in the vector and flanking the inserts and verified by Sanger sequencing. PureLink HiPure midiprep kit (Invitrogen) was used to extract plasmids from 100 ml of bacterial overnight cultures grown in LB medium supplemented with 25 μg/ml of kanamycin.

#### Expression of Fusion Proteins and LIPS Assay

A modified version of Burbelo's protocol was developed for expression of fusion proteins: HEK-293A cells (kindly provided by Bernard Klonjkowski, Veterinary School, Maisons-Alfort, France) were transfected with polyethylenimine (PEI, Polyscience Inc., USA) instead of Cos-1 cells and Fugene-6 transfecting reagent (Burbelo et al., 2007, 2009). Cells were maintained in Dulbecco's modified Eagle's medium supplemented with 10% fetal calf serum, 1% sodium pyruvate, 1% penicillin-streptomycin mix, 1% amphotericin B, and 1% non-essential amino acids (Invitrogen) with two passages a week. Cells (4 × 10^5^ per well) were plated on a six-well plate the day before transfection, and 5 μg of plasmid DNA was transfected with 20 μl of 1 mg/ml PEI. Two days post-transfection, fusion proteins were harvested as previously described (Burbelo et al., 2007, 2009). Protease inhibitors (complete protease inhibitor cocktail minitablets, Roche Applied Science) and 10% of glycerol were added to the cell lysates to enhance protein stability. The luciferase activity of the fusion proteins was measured onto a Centro XS^3^ LB 960 luminometer (Berthold Technologies, France) by adding the NanoLuc substrate (NanoGlo reagent, Promega) to 10-fold dilutions of the lysates. Titers (in light unit LU/ml) were corrected for background by subtracting LU values produced by negative controls constituted of cells transfected with the same amount of PEI without plasmid DNA.

LIPS assay was performed as previously described by Burbelo et al. (2007, 2009) except that human and cattle sera were not diluted. Sera of 30 healthy French donors living in Paris area who did not report travel to Thailand or any tick bite (kindly provided by ICAReB platform of Institut Pasteur, Paris, France) were screened for the presence of antibodies against the targeted viruses as a non-exposed group control. Residual background was measured as the mean of 10 negative controls (without serum), and positivity threshold was defined as the mean of these controls + 5 standard deviations.

### Statistical Analyses

Significant differences between exposed and non-exposed groups of human sera tested by LIPS were calculated using the Student *t*-test (95% confidence interval). The maximal prevalence of viral infection in each exposed group was estimated assuming a sensitivity of 90% and a specificity of 99% of the test and the Blaker confidence interval calculation using the True Prevalence calculator implemented in the EpiTools portal (https://epitools.ausvet.io/trueprevalence).

### Accessions Numbers

Complete coding regions of the six viruses characterized were deposited into the GenBank database under the numbers MN095535 to MN095546.

## Results

### The Viral Transcriptome of Thai Ticks Is Mainly Constituted of Negative-Strand ssRNA Viruses

The transcriptome of Thai ticks was sequenced at a depth of 222 × 10^6^ reads. Most sequences were assigned to the Eukaryota domain while bacteria and viruses represented 0.09 and 0.001% of validated taxonomic assignation of reads, respectively. Of these, more than 99% of viral sequences were composed of RNA viruses, while few other related viral sequences were identified (Table 1). ssRNA negative-strand viruses represented 97.20% of all viruses, followed by ssRNA positive strand viruses (1.94%), unclassified viruses (0.70%), and dsRNA viruses (0.14%). Only few sequences were assigned to DNA viruses (ssDNA: 0.01%), reflecting the specificity of RNA extraction. The identification of ssDNA viruses would more likely correspond to the sequencing of ssDNA viral transcripts, reflecting the replicative form of these viruses.

**Table 1:** Viral sequences identified in ticks from Thailand by high-throughput sequencing.

	**Order**	**Family**	**Genus**	**Type of species**	**Abundance**	**AA identity**
ssRNA-	*Mononegavirales*	*Rhabdoviridae*	Unclassified	Rhipicephalus associated rhabdo-like virus*	55,722	93–97%
				Tacheng tick virus 3	10,941	53–81%
				Nayun tick rhabdovirus	9,632	99%
				American dog tick rhabdovirus-2	225	65–84%
				Eelpout rhabdovirus	66	75–81%
				Bole tick virus 2	65	68–72%
				Wuhan redfin culter dimarhabodovirus	64	81–83%
				Taishun tick virus	62	63–69%
			*Sprivivirus*	Carp sprivivirus	62	76%
			*Vesiculovirus*	Maraba virus	60	75%
			*Ledantevirus*	Nkolbisson virus	58	77%
		*Chuviridae*	*Mivirus*	Wuhan tick virus 2*	291,705	89–92%
				Brown dog tick mivirus 1*	3,415	96–99%
				Bole tick virus 3	158	60–84%
		Unclassified	Unclassified	Norway mononegavirus 1	29,576	55–64%
	*Bunyavirales*	*Phenuiviridae*	*Phlebovirus*	*Rhipicephalus* associated phlebovirus 1*	125,363	97–100%
				Tick phlebovirus Anatolia 1	7,397	83–100%
				American dog tick phlebovirus	801	51–96%
				Tacheng tick virus 2	607	60–92%
				Changping tick virus 1	516	66–90%
				Pacific coast tick phlebovirus	218	60–96%
	*Articulavirales*	*Orthomyxoviridae*	*Thogotovirus*	Oz virus*	56,038	70–82%
				Dhori thogotovirus	345	82%
			*Quaranjavirus*	Zambezi tick virus 1	121	84–93%
				Wellfleet Bay virus	65	71–72%
ssRNA+	N/A	Unclassified	Unclassified	Trinbago virus*	11,643	93%
		*Flaviviridae*	*Hepacivirus*	Bovine hepacivirus	99	93–97%
			*Pestivirus*	Pestivirus H	62	100%
		*Luteoviridae*	Unclassified	Norway luteo-like virus 2	33	72%
dsRNA	N/A	*Reoviridae*	*Orbivirus*	St Croix River virus	773	72–100%
				Wad Medani virus	65	81–90%
		*Totiviridae*	Unclassified	Lonestar tick totivirus	32	71%
ssDNA	N/A	*Parvoviridae*	Copiparvovirus	Bovine parvovirus-2	84	87–92%
Unclassified	Unclassified	Unclassified	Unclassified	Tick-borne tetravirus-like virus	4,292	60–93%

*Sequences that were individually verified for the absence of endogenous viral elements are highlighted with an asterisk*.

Among the ssRNA viruses, positive-strand RNA viruses were in the minority (1.96% of ssRNA viruses). More than 98% of sequences were taxonomically related to Trinbago virus, a BLTV4-related virus identified in Trinidad and Tobago *Rh. sanguineus* ticks (Sameroff et al., 2019). The strain identified in Thai ticks, tentatively named BLTV4-Thailand virus (accession no. MN095535) presented a mean amino-acid identity of 93% with the polyprotein of Trinbago virus (Table 1). Other positive-strand ssRNA viruses were less frequently identified and were assigned to the *Hepacivirus* and *Pestivirus* genera (more likely corresponding to cattle-infecting viruses that are detected through the bloodmeal of ticks), or to the *Luteoviridae* family (Table 1).

The negative-strand ssRNA viruses constituted more than 97% of viral sequences (Table 1, Supplemental Figure 1). Among these, more than 66% of ssRNA viruses were assigned to the nonsegmented *Mononegavirales* or *Jingchuvirales* orders. For example, sequences assigned to the *Rhabdoviridae* family mainly mapped onto a novel strain of Wuhan tick virus 1 (WhTV1), with amino-acid identities ranging from 93 to 97%, depending on the gene (WhTV1-Thailand virus, accession no MN095536); while other unclassified *Rhabdoviridae*-related sequences were detected (as for example a distant Tacheng tick virus 3 virus or a new strain of Nayun tick rhabdovirus). Few sequences, were assigned to the *Sprivivirus*, the *Vesiculovirus*, and the *Ledantevirus Rhabdoviridae* genera. Viruses belonging to the *Chuviridae* mapped onto Wuhan tick virus 2 (WhTV2) and Brown dog mivirus 1 (a new strain of Changping tick virus 2 [CpTV2]), with amino-acid identities ranging from 89 to 99% (WhTV2-Thailand virus: MN095546; and CpTV2-Thailand virus: MN095545). Finally, a novel unclassified mononegavirus genome presented distant homologies with Norway mononegavirus 1 (amino-acid identities ranging from 55 to 64%, depending on the gene).

Up to 23% of ssRNA viruses were assigned to the *Bunyavirales*, and more precisely to the *Phenuiviridae* family. More than 92% of sequences were related to Rhipicephalus-associated phlebovirus 1, a novel strain of Lihan tick phlebovirus (LTPV) primarily associated with *Rh. microplus* Chinese ticks. Tentatively named LTPV-Thailand virus, this viral genome presented amino-acid identities ranging from 97 to 100% with its closest viral relative, depending on the gene (accession no MN095537-38). Several sequences close to Tick phlebovirus Anatolia 1 were also detected.

Finally, *Orthomyxoviridae*-related sequences (more than 9% of total ssRNA viruses), either belonging to the *Thogotovirus* or the *Quaranjavirus* genera were detected, but Oz virus-related viral sequences were the most abundant. Tentatively named Thailand tick thogotovirus (accession no MN095539-44), this virus presented amino-acid identities ranging from 70 to 82% depending on the genes (Table 1) with Oz virus, a recently reported tick-borne virus identified in *Amblyomma testudinarium* ticks from Japan that is phylogenetically close to Bourbon and Dhori thogotoviruses (Ejiri et al., 2018).

For each strain of BLTV4-Thailand virus, WhTV1-Thailand virus, WhTV2-Thailand virus, CpTV2-Thailand virus, LTPV-Thailand virus, and Thailand tick thogotovirus described hereafter, the presence of the virus in the initial RNA pool used to produce HTS library was validated by specific reverse transcription PCR targeting different portions of the genome (data not shown).

### Genomic Organization and Phylogenetic Analyses of *Rhabdoviridae*-, *Chuviridae*-, *Phenuiviridae*-, *Flaviviridae*-, and *Orthomyxoviridae*-Related Viruses

#### *Rhabdoviridae*/WhTV1-Thailand Virus

**Figure 1:**
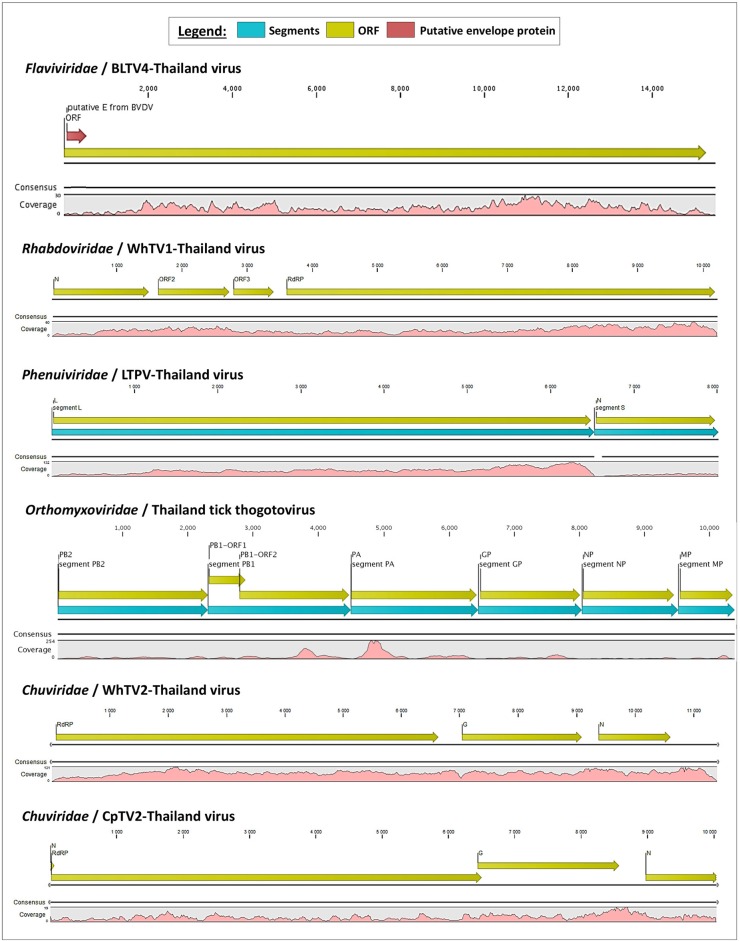
Schematic organization of the six viral genomes identified in Thai ticks. The open reading frames (ORFs) are indicated with yellow arrows, and genome coverage is indicated in pink. Segmented viruses were presented as concatenated sequences for better clarity (blue arrows represent the different segments). The putative envelope glycoprotein (GP) of Thailand tick flavivirus is highlighted by a red arrow in the polyprotein ORF.

*Rhabdoviridae* is a family of RNA viruses infecting a large spectrum of hosts, ranging from plants to invertebrates and vertebrates. Tick-borne rhabdoviruses include viruses belonging to the recognized *Ledantevirus* and *Ephemerovirus*, and the putative *Sawgravirus* genera (Labuda and Nuttall, 2004; Walker et al., 2015; Tokarz et al., 2018). *Rhabdoviridae* viruses present a general genome organization of linear negative-strand ssRNA genome usually encoding five proteins, in the order N (nucleoprotein)/P (phosphoprotein)/M (matrix)/G (glycoprotein)/L (RNA-dependent RNA polymerase). WhTV1-Thailand virus presents a similar gene organization, although only four ORFs were identified (Figure 1). ORF1 code for a protein of 488 aa corresponds to the putative nucleoprotein of the virus. ORF2 code for a hypothetical protein of 363 aa presenting numerous O-glycosylation sites. Its size (in the range of those observed for other *Rhabdoviridae* phosphoproteins), the absence of transmembrane domains, and its richness in acidic amino acids (PI = 4.89) suggest that ORF2 could code for the putative P protein of WhTV1-Thailand virus. ORF3 codes for a small protein of 205 aa corresponding to the putative matrix protein of the virus. The last ORF codes for a large protein of 2,183 aa corresponding to the RNA polymerase of the virus. WhTV1-Thailand virus present therefore an N–P–M–L gene organization, suggesting that a major evolution event in its emergence was the loss of G gene. Walker et al. noted that gain and/or loss of genes was common and a major driver of rhabdoviruses evolution (Walker et al., 2015), and several assumptions were made to explain the mechanism of entry in hosts cells of such viruses presenting the loss of G protein, as for example the use of a helper virus (Sameroff et al., 2019). WhTV1-Thailand viral ORFs present genetic identity ranging from 93 to 97% at the protein level, depending on the gene, with its closest viral relative *Rhipicephalus* associated rhabdo-like virus (Table 2).

**Table 2:** Amino-acid identity of *Flaviviridae*-, *Rhabdoviridae*-, *Chuviridae*-, *Phenuiviridae*-, and *Orthomyxoviridae*-related viruses with their closest viral reference genome.

**Virus**	**Gene**	**Closest relative**	**% identity (aa)**	**Tick species**	**Prevalence**
WhTV1-Thailand (WhTV1-T)	N	*Rhipicephalus*-associated rhabdo-like virus (*Rh. microplus*/China/2016)	96.72%	*Rhipicephalus microplus*	21.68%
	ORF2		92.84%		
	ORF3		95.07%		
	L		97.39%		
WhTV2-Thailand (WhTV2-T)	L	Wuhan tick virus 2 (*Rh. microplus*/China/2012)	92.28%	*Rhipicephalus microplus*	18.18%
	G	Wuhan tick virus 2 (*Rh. microplus*/Trinidad and Tobago/2017)	90.71%		
	N		88.56%		
CpTV2-Thailand (CpTV2-T)	L	Brown dog tick mivirus 1 (*Rh. sanguineus*/Trinidad and Tobago/2017)	99.17%	*Rhipicephalus sanguineus*	18.75%
	G		97.62%		
	N		96.50%		
LTPV-Thailand (LTPV-T)	L	*Rhipicephalus* associated phlebovirus 1 (*Rh. microplus*/China/2016)	99.67%	*Rhipicephalus microplus*	20.98%
	S		97.48%		
BLTV4-Thailand (BLTV4-T)	Polyprotein	Trinbago virus (*Rh. sanguineus*/Trinidad and Tobago/2017)	92.79%	*Rhipicephalus sanguineus*/*microplus*	18.75/0.70%
Thailand tick thogotovirus (TT-THOV)	PB2	Oz virus (*Am. testudinarium*/Japan/2013)	70.79%	*Rhipicephalus microplus*	2.80%
	PB1-ORF1		80.14%		
	PB1-ORF2		78.32%		
	PA		69.86%		
	GP		68.58%		
	NP		82.07%		
	M		78.52%		

*The tick species and global prevalence of detection are indicated*.

**Figure 2:**
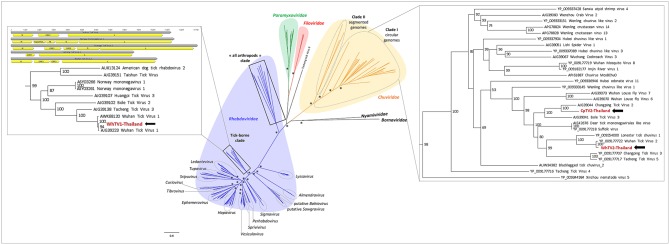
Phylogenetic relationship of *Mononegavirales*- and *Jingchuvirales*-related viral genomes identified in Thai ticks with other representative viruses. Nodes with bootstrap values >50 are noted with an asterisk. Phylogenetic reconstruction was performed by maximum likelihood on the complete RNA-dependent RNA polymerase (RdRP) amino-acid gene (model: LG + G + I + F).

Recently, numerous tick-associated rhabdoviruses were reported worldwide, but they are still unassigned to a genus and fall in several distinct clades that possibly constitute new genera in the family (Tokarz et al., 2014b; Li C. X. et al., 2015; Xia et al., 2015; Brinkmann et al., 2018). Phylogenetic analyses performed on the complete amino-acid sequence of the RNA-dependent RNA polymerase revealed that WhTV1-Thailand virus falls in a clade, distant from classical rhabdoviruses, that comprised viruses identified exclusively in ticks from a wide range of geographical origins (China, USA, Turkey and Norway) (Figure 2). One should note that the genome organization of several viruses within this subclade of tick-borne viruses (comprising WhTV1-Thailand virus) present also gene loss (Figure 2, left inset).

#### *Chuviridae*/WhTV2- and CpTV2-Thailand Viruses

*Chuviridae*, a recently recognized viral family among the *Jingchuvirales* that was primarily assigned to the *Mononegavirales* order forms a monophyletic group of segmented, non-segmented, and circular viruses (Siddell et al., 2019) at median distance with segmented and unsegmented negative-strand RNA viruses (Li C. X. et al., 2015). They infect a wide variety of invertebrate hosts, including Crustacea, Nematoda, Insecta, Myriapoda, Arachnida, and Ixodida. Among tick-borne chuviruses, both *Argasidae* (Argas mivirus) and *Ixodidae* ticks are infected by tick-borne chuviruses (e.g., Bole, Changping, Dermacentor, Lonestar, Suffolk, and Wuhan mivirus). *Chuviridae* viruses present a large variety of genome organization, from a circular L–G–N order of genes (clade I, Figure 2) to segmented or linear G–N–L order of genes (clade II, Figure 2) (Li C. X. et al., 2015). WhTV2-Thailand virus present a genome of 11.4 kb with three ORFs: the first ORF codes for a large protein of 2,189 aa corresponding to the RNA-dependent RNA polymerase of the virus; ORF2 code for the putative glycoprotein of 683 aa and ORF3 codes for the nucleoprotein of 411 aa (Figure 1). WhTV2-Thailand virus genome was confirmed to belong to the circular L–G–N form of *Chuviridae* by performing PCR with the forward primer located in 3′ of the genome and the reverse primer in the 5′ part (data not shown). CpTV2-Thailand virus presents similar genome organization as WhTV2-Thailand virus, with a circular ssRNA genome, except that its genome is shorter (10 kb) and that the G ORF overlaps the L ORF on 61 nucleotides (this junction was confirmed by specific amplification followed by Sanger sequencing). The L gene code similarly for the RdRP of 2,164 aa, the G gene codes for the glycoprotein of 710 aa and the N gene codes for the nucleoprotein of 374 aa (Figure 1).

WhTV2-Thailand virus presents amino acid identity ranging from 89 to 92% with the prototype Wuhan tick virus 2 while CpTV2-Thailand virus is close to Brown dog tick mivirus 1, a Changping tick virus 2-related virus (Table 2). Phylogenetic analyses performed on the complete amino-acid sequence of the RdRP confirmed that WhTV2-Thailand and CpTV2-Thailand chuviruses belong to the circular clade I of the *Chuviridae* (Figure 2). In addition, with a highly supported bootstrap of 100, they both placed in a subclade composed exclusively of viruses identified in ticks (Figure 2, right inset).

#### *Phenuiviridae*/LTPV-Thailand Virus

*Phenuiviridae* is a family of segmented negative-strand ssRNA viruses which comprise *Goukovirus* and *Phasivirus* (insect-specific viruses), *Tenuivirus* (plant arboviruses), and *Phlebovirus* (animal arboviruses) genera. This latter is the unique known viral genus among the family able to infect vertebrates, including humans and domestic animals. Recognized tick-borne phleboviruses (TBPVs) are currently clustered into three main serogroups: SFTS virus, Bhanja virus, and Uukuniemi virus serogroups (Yu et al., 2011; Matsuno et al., 2013; Palacios et al., 2013). The ecological cycle of TBPVs implies *Ixodidae* ticks as the main vector, wild small animals (e.g., *Soricidae* or *Erinaceidae*) or migratory birds (Li et al., 2016) as putative reservoirs and/or amplifying hosts (also responsible of the dissemination of the virus over long distances), and accidental mammalian hosts. SFTS-like viruses are able to infect humans, while other TBPVs usually infect domestic animals like sheep, goat, and cattle (Hubálek et al., 2014), and, in rare specific cases, humans (Calisher and Goodpasture, 1975). Viruses belonging to this genus are trisegmented, with L segment coding for the viral RdRP, M segment coding for the glycoprotein precursor which will be further matured in two glycoproteins (GP) and a non-structural protein (NS), and the S segment coding for the nucleoprotein (NP) and a NS protein. Recently, bi-segmented phlebovirus-like viruses without any M segment were reported in ticks from various origins (Li C. X. et al., 2015). LTPV-Thailand virus presents the same bisegmented genome architecture, with L and S segments of 6,517 and 1,406 nt, respectively (which is in the range of trisegmented phleboviruses), coding for the expected RdRP and NP genes. As for other bisegmented phleboviruses, LTPV-Thailand virus lacked the M segment. It has been proposed that a high degree of divergence of M segments of these phleboviruses may explain that usual Blast-based methods of taxonomic assignation of reads have missed this segment (Li C. X. et al., 2015). As a consequence, the only identified structural protein of LTPV-Thailand virus was NP, which interestingly present structural homologies with the nucleoprotein of SFTS virus, a tick-borne phlebovirus causing severe symptoms in humans and close to the Uukuniemi virus serocomplex (Yu et al., 2011).

**Figure 3:**
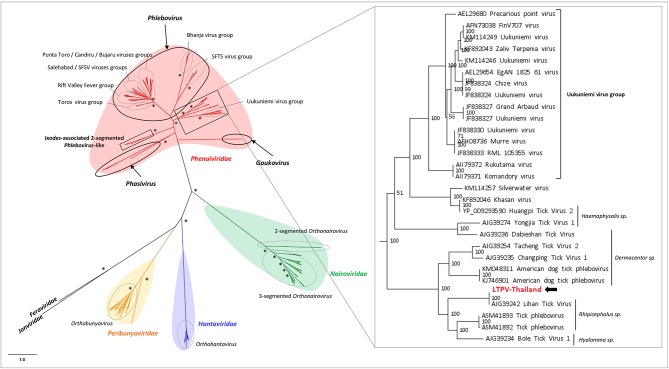
Phylogenetic relationship of *Bunyavirales*-related viral genomes identified in Thai ticks with other representative viruses. Nodes with bootstrap values >50 are noted with an asterisk. Phylogenetic reconstruction was performed by maximum likelihood on the complete RNA-dependent RNA polymerase (RdRP) amino-acid gene (model: LG + G + I + F).

LTPV-Thailand virus presents amino acid identity of 97 and 100% with Rhipicephalus-associated phlebovirus 1, a new strain of Lihan tick phlebovirus identified in *Rh. microplus* Chinese ticks (Table 2). Phylogenetic analyses performed on the complete RdRP amino acid gene revealed that LTPV-Thailand virus is located in a clade at the root of the Uukuniemi virus group, a tick-borne phlebovirus primarily isolated in cattle-infesting *Ixodes ricinus* ticks from Finland that could sporadically infect cattle and humans (Saikku, 1973; Traavik and Mehl, 1975) (Figure 3). Two subclades of bisegmented phlebovirus-like viruses are placed at the root of UUKV, one only composed of tick-borne viruses identified in *Ixodes* (*I. ricinus* and *I. scapularis*) ticks from USA and Norway (Pettersson et al., 2017; Tokarz et al., 2018), while the other (containing LTPV-Thailand virus) is formed by viruses detected in *Rhipicephalus, Dermacentor, Haemaphysalis*, and *Hyalomma* ticks from China, Brazil, USA and Turkey (Tokarz et al., 2014a; Li C. X. et al., 2015; Dinçer et al., 2017; Brinkmann et al., 2018; Souza et al., 2018), suggesting a possible association between the tick species and the virus and a putative coevolution of bisegmented phleboviruses with their respective tick host (Figure 3, inset).

#### *Flaviviridae*/BLTV4-Thailand Virus

*Flaviviridae* is a family of positive-strand ssRNA viruses composed of four recognized genera: *Flavivirus* (arboviruses infecting both vertebrates and invertebrates), *Pegivirus* (mammalian viruses including primates, humans, pigs, and bats), *Pestivirus* (animal viruses such as bovine diarrhea virus or classical swine fever virus), and *Hepacivirus* (human Hepatitis C virus). Tick-borne flaviviruses (TBFVs) are divided into mammalian- and seabird-infecting viruses (Brackney and Armstrong, 2016). Mammalian TBFVs are transmitted by *Ixodidae* hard ticks, while seabird TBFVs are transmitted by *Argasidae* soft ticks. The flavivirus viral genome of ~10–12 kb codes for a unique polyprotein that will be cleaved during maturation by viral and host proteases into viral structural and non-structural proteins. Recently, Shi et al. reported the identification of divergent flavivirus-like viruses in arthropods with genome size ranging from 16 to 26 kb with similar genome organizations, including Bole tick virus 4 (BLTV4) virus primarily identified in *Hy. asiaticum* Chinese ticks and later in *Rh. sanguineus* ticks from Trinidad and Tobago (Trinbago virus) (Shi et al., 2015; Sameroff et al., 2019). We identified in Thai ticks a genome close to Trinbago virus, tentatively named BLTV4-Thailand virus. Its genome of 15.5 kb (shorter than BLTV4 genome of 16.2 kb) codes for a unique polyprotein of 5,089 aa.

**Figure 4:**
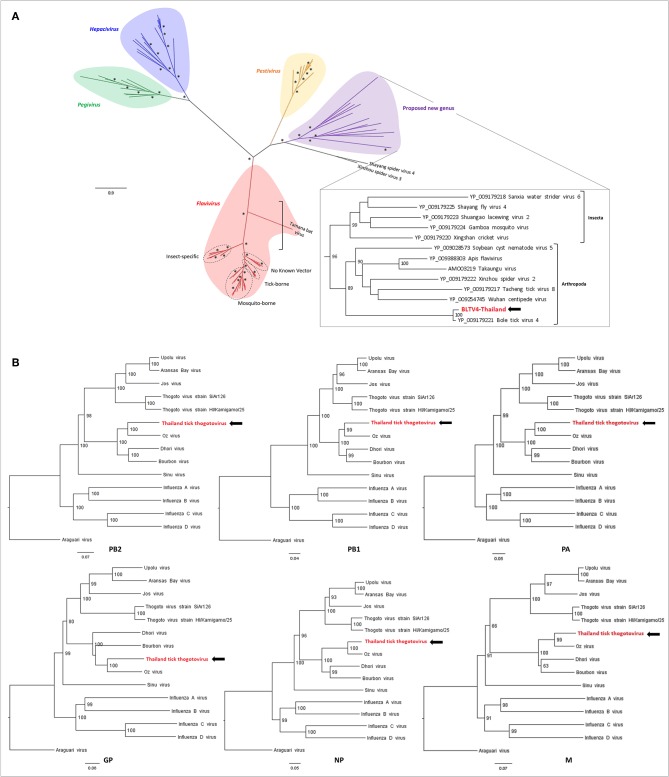
Phylogenetic relationship of viral genomes identified in Thai ticks with other representative viruses. **(A)** Maximum likelihood (ML) tree of the complete RNA-dependent RNA polymerase (RdRP) amino-acid sequence of representative *Flaviviridae* viruses (model: LG + G + I + F). Nodes with bootstrap values >50 are noted with an asterisk. **(B)** Neighbor joining (NJ) trees of the complete PB2–PB1–PA–GP–NP–M amino-acid sequences of representative *Orthomyxoviridae* viruses (model: *p*-distance).

Phylogenetic analyses performed on the RdRP region of representative sequences of *Flaviviridae* placed BLTV4-Thailand virus in a sister clade of the *Pestivirus* genus that contains viruses only identified in invertebrates, as previously described (Sameroff et al., 2019). Putatively forming the fifth genus of the *Flaviviridae* family, this group of viruses infecting arthropods placed at the root of the four recognized genera (Figure 4A), supporting the hypothesis of Shi et al. that flaviviruses may originate from arthropods (Shi et al., 2015). Interestingly, with a highly supported node of 96, two subclades compose this putative genus: one restricted to the class of Insecta and the second, including BLTV4-Thailand virus, composed of viruses infecting all types of arthropods, ranging from Chelicerata to Myriapoda and Hexapoda, suggesting a possible more ancestral position of this virus in the evolution of flaviviruses (Figure 4A, inset) and a later divergence of insect-specific *Flaviviridae*-related viruses.

#### *Orthomyxoviridae*/Thailand Tick Thogotovirus

The *Orthomyxoviridae* family is composed of seven genera: types A/B/C and D *Influenzavirus, Isavirus, Quaranjavirus*, and *Thogotovirus*. Thogotoviruses, arthropod-borne viruses infecting mammals mainly transmitted by ticks, have been identified in Africa, North America, Southern Europe, the Middle East, and in Far East Asia, either in ticks and/or in humans (for Thogoto virus, Dhori virus, and Bourbon virus) (Ejiri et al., 2018). Thogotoviruses are composed of six negative-strand ssRNA segments that code from the polymerase complex (segments PB2, PB1, and PA), the glycoprotein (GP), the nucleoprotein (NP) and the matrix protein (M). Although influenza viruses present a surface GP constituted of hemagglutin and neuraminidase proteins, the GP of thogotoviruses present structural similarities with the gp64 envelope glycoprotein of alphabaculoviruses (Morse et al., 1992; Kadlec et al., 2008). We identified in Thai ticks a viral genome close to Oz virus (OZV), a thogotovirus recently isolated from *Am. testudinarium* ticks in Japan (Ejiri et al., 2018). Provisionally named Thailand tick thogotovirus, this virus presents the characteristics of a *Thogotovirus* member, meaning: (i) the six segments genome architecture [segment 1 codes for the putative PB2 protein of 764 aa; segment 2 for two ORFs (of 187 and 559 aa, respectively)] constituting the PB1 protein complex; segment 3 for the PA protein of 637 aa; segment 4 for the GP protein of 510 aa; segment 5 for the NP protein of 465 aa; and segment 6 for the M protein of 252 aa); and (ii) the structural conformation of Thailand tick thogotovirus GP close to the baculovirus fusion protein gp64 (data not shown) (Morse et al., 1992; Kadlec et al., 2008). Thailand tick thogotovirus presents an amino-acid identity with OZV ranging from 69% (GP) to 82% (NP) (Table 2).

Phylogenetic analyses performed on the six segments of Thailand tick thogotovirus and representative *Thogotovirus* genomes resulted in the same topology, i.e., a clustering of Thailand tick thogotovirus with OZV in a sister clade of the group formed by Bourbon and Dhori viruses, suggesting that no reassortment events occurred during Thailand tick thogotovirus evolution (Figure 4B).

### Prevalence of *Flaviviridae-, Rhabdoviridae-, Chuviridae-, Phenuiviridae*-, and *Orthomyxoviridae*-Related Viruses

Among the 266 tick extracts used to generate the pool sequenced, 231 were available and screened individually for the presence of Thailand tick thogotovirus and other LTPV-, BLTV4-, WhTV1-, WhTV2-, and CpTV2-Thailand viruses. Results are presented in Table 3A. LTPV-Thailand phlebovirus was only detected in *Rh. microplus* ticks at a global prevalence of 21%. BLTV4-Thailand virus was mainly detected in *Rh. sanguineus* ticks (19%) and in one *Rh. microplus* tick. Interestingly, these two viruses are genetically close but presented only 82% of nucleotide identity in the RdRP gene. Thogotovirus-related virus was identified at low prevalence (<3%) in adult *Rh. microplus* ticks, while WhTV2-Thailand chuvirus was more prevalent (18%). CpTV2-Thailand virus was only detected in *Rh. sanguineus* ticks at a global prevalence of nearly 19%. Finally, WhTV1-Thailand virus was only detected in more than 21% of *Rh. microplus* ticks (Tables 2, 3A).

**Table 3:** Results of prevalence studies of *Flaviviridae*-, *Rhabdoviridae*-, *Chuviridae*-, *Phenuiviridae*-, and *Orthomyxoviridae*-related viruses.

**A**.					**Virus detection (Nb-positive ticks)**					
**Tick genus/** **species**	**Stage/** **Gender**	**Nb** **tested**	**Collected** **on**	**Bloodmeal**	**LTPV-** **Thailand**	**BLTV4-** **Thailand**	**Thailand tick** **thogotovirus**	**WhTV2-** **Thailand**	**CpTV2-** **Thailand**	**WhTV1-** **Thailand**
*Amblyomma* sp.	Adult F	11	Wild boar	nd	0	0	0	0	0	0
	Adult F	8	Wild boar	+	0	0	0	0	0	0
	Adult M	1	Rodent	+	0	0	0	0	0	0
*Rhipicephalus microplus*	Adult F	136	Cattle	+	29	1	4	25	0	30
	Adult F	1	Flagging	nd	0	0	0	0	0	0
	Adult M	2	Cattle	+	0	0	0	0	0	0
	Adult M	1	Flagging	nd	0	0	0	0	0	0
	Adult M	1	Cattle	nd	0	0	0	0	0	0
	Nymph	1	Cattle	+	1	0	0	1	0	1
	Nymph	1	Cattle	nd	0	0	0	0	0	0
*Dermacentor marginatus*	Adult F	11	Wild boar	nd	0	0	0	0	0	0
	Adult M	4	Wild boar	nd	0	0	0	0	0	0
	Nymph	1	Flagging	nd	0	0	0	0	0	0
*Haemaphysalis hystricis*	Adult F	10	Wild boar	nd	0	0	0	0	0	0
	Adult M	3	Wild boar	nd	0	0	0	0	0	0
	Nymph	2	Dog	nd	0	0	0	0	0	0
	Nymph	3	Flagging	nd	0	0	0	0	0	0
*Hyalomma* sp.	Adult F	8	Wild boar	nd	0	0	0	0	0	0
	Adult F	7	Wild boar	+	0	0	0	0	0	0
	Adult F	1	Dog	+	0	0	0	0	0	0
	Nymph	2	Dog	+	0	0	0	0	0	0
*Rhipicephalus sanguineus*	Adult F	4	Dog	nd	0	1	0	0	2	0
	Adult M	4	Dog	nd	0	1	0	0	1	0
	Nymph	8	Dog	nd	0	1	0	0	0	0
**B**.		**LTPV-T**		**BLTV4-T**		**Thogotovirus**	**WhTV2-T**		**CpTV2-T**	**WhTV1-T**
LTPV-Thailand		–								
BLTV4-Thailand		nd		–						
Thailand tick thogotovirus		nd		nd		–				
WhTV2-Thailand		1 (*Rh. microplus*)		nd		nd	–			
CpTV2-Thailand		nd		2 (*Rh. sanguineus*)		nd			–	
WhTV1-Thailand		6 (*Rh. microplus*)		nd		nd	6 (*Rh. microplus*)		nd	–

*(A) Prevalence of positive ticks. (B) Matrix of coinfections. nd: not detectable*.

Few coinfections of ticks were detected. The presence of two viruses (most frequently WhTV1-Thailand virus associated with LTPV-Thailand virus or WhTV2-Thailand virus) in the same individual tick was identified in 13 *Rh. microplus* and in 2 *Rh. sanguineus* ticks (Table 3B). Four coinfections with three different viruses were also detected in *Rh. microplus* ticks (three coinfections LTPV-/WhTV1-/WhTV2-viruses, and one coinfection LTPV-/thogoto-/WhTV2-viruses).

Ticks harbor numerous EVEs within their genome (Katzourakis and Gifford, 2010; Li C. X. et al., 2015), including several sequences phylogenetically related to viruses identified in the present study. Therefore, ticks positive either for LTPV-, BLTV4-, WhTV1-, WhTV2-, CpTV2-, and thogoto-Thailand viruses were individually screened for the presence of EVE using their corresponding DNA extract. No positive result was obtained, showing that the viruses identified by next generation sequencing are exogenous viruses (data not shown).

### LIPS-Based Serological Screening of Human and Cattle Sera

**Figure 5:**
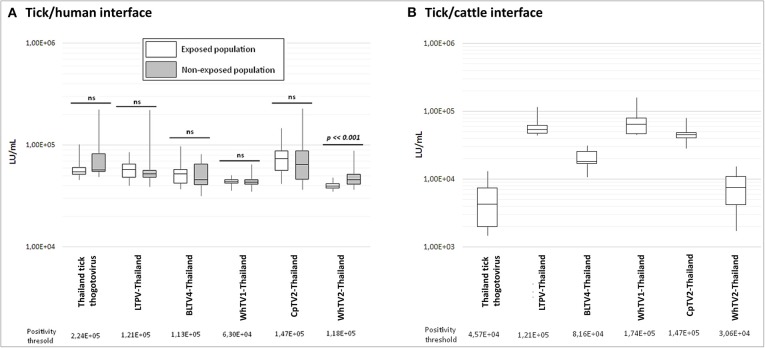
Luciferase activity (in LU/ml) distribution of measures after luciferase immunoprecipitation system (LIPS) performed in **(A)** tick/human interface and **(B)** tick/cattle interface. In white: human and cattle populations exposed to tick bites; in gray: non-exposed human populations. Positivity threshold is indicated for each antigen construct. *t*-test statistical analysis (α = 0.05) was used to compare the mean LU/ml measure of both exposed and non-exposed human groups.

To test if one or more viruses identified among Thai ticks is able to infect selected mammalian species (humans and cattle) highly exposed to tick bites, and therefore could constitute putative novel tick-borne arboviruses, we developed LIPS-based serological screening against Thailand tick thogotovirus and LTPV-, BLTV4-, WhTV1-, WhTV2-, and CpTV2-Thailand viruses. Results are presented in Figure 5.

For the tick/human interface, no significant difference was observed between exposed and non-exposed groups (Figure 5A), and no serum exceeded the positivity threshold, except for WhTV2-Thailand chuvirus antigen, which presents a higher luciferase activity in the non-exposed group than in the exposed group (*p* = 0.0003), while none of the sera in the exposed nor non-exposed groups exceeded the positivity threshold. Similarly, none of the cattle sera presented a luciferase activity higher than the positivity threshold (Figure 5B), showing that no antibodies against one of the six viruses tested were detected in human and cattle sera. The maximal prevalence of sera reacting with at least one of the six viral constructs was estimated at 0.017% and 0.047% (*p* = 0.05) in humans and cattle, respectively, meaning that these infections of vertebrates, if they occur, are very rare.

## Discussion

The increasing accessibility of HTS technology and the resulting description of viral communities in various environmental samples pave the way for a better understanding of viral spillovers. However, identifying a list of viruses at higher risk of emergence requires first the characterization of the ecological cycle of the virus, including its putative reservoir hosts, hematophagous arthropod vectors (in the case of arboviruses), and vertebrate recipient hosts (Temmam et al., 2014). Usual practices combine field surveillance of vectors and wildlife, description of viral communities present in these animals, analyses of bloodmeal origin in arthropods, and phylogenetic analyses of viruses. Recently, novel approaches tried to predict vertebrate and invertebrate hosts using computational modeling (Babayan et al., 2018). In this study, we combined field surveillance of arthropods and virome characterization with comprehensive phylogenetic analyses to select relevant viruses close to tick-borne arboviruses. We further characterized putative spillover events by applying a systematic serological survey in human and cattle populations highly exposed to these ticks. Among the viral communities characterized in Thai ticks, we selected viruses belonging to four viral families known to contain zoonotic viruses (*Orthomyxoviridae, Phenuiviridae, Rhabdoviridae*, and *Flaviviridae* families) and a recently recognized viral family for which the zoonotic potential was still unknown (*Chuviridae*).

The *Phenuiviridae, Flaviviridae, Orthomyxoviridae, Rhabdoviridae*, and *Chuviridae* viruses detected in *Rh. microplus* and *Rh. sanguineus* Thai ticks were previously reported in other ticks species and genera, highlighting a low degree of host restriction and an increase in the vector host range of these viruses (Table 4). For example, CpTV2-Thailand chuvirus identified in *Rh. sanguineus* was previously reported in Chinese *Dermacentor* sp. and *Ha. parva* ticks (Li C. X. et al., 2015), in Turkish *Ha. parva* ticks (Brinkmann et al., 2018), and in *Rh. sanguineus* ticks from Trinidad and Tobago (Sameroff et al., 2019). Similarly, Thailand tick thogotovirus, identified in *Rh. microplus* ticks, is phylogenetically related to Oz virus isolated in Japan from *Am. testudinarium* tick (Ejiri et al., 2018). LTPV-Thailand phlebovirus presented also a low degree of host specificity, as regards to its reports in Thai *Rh. microplus* ticks, in *Dermacentor nitens* from Colombia, in *Rh. microplus* from China (Li C. X. et al., 2015), Brazil (Souza et al., 2018), and Trinidad and Tobago (Sameroff et al., 2019), and in *Hy. marginatum* (Dinçer et al., 2017) and *Rh. sanguineus* (Brinkmann et al., 2018) from Turkey. Finally, BLTV4-Thailand flavivirus, identified in Thai *Rh. microplus* and *Rh. sanguineus* ticks, was either previously reported in *Hy. asiaticum* (Shi et al., 2015), *Hyalomma detritum*, and *D. marginatus* ticks from China, and in *Rh. sanguineus* ticks from Trinidad and Tobago (Sameroff et al., 2019). This low degree of host restriction [either suggesting multiple hosts switches or reflecting the non-viremic transmission of viruses occurring during cofeeding onto the same host (Labuda and Nuttall, 2004)], added to the phylogenetic placement of these viruses at the root of known tick-borne arboviruses, raised the question of their possible ability to infect vertebrate hosts.

**Table 4:** Tick spectrum and geographical origin of *Flaviviridae*-, *Rhabdoviridae*-, *Chuviridae*-, and *Phenuiviridae*-related viruses.

**Virus**	**Tick species**	**Country**	**Reference**
Bole tick virus 4 (BLTV4)	*Rhipicephalus microplus*	Thailand	This study
	*Rhipicephalus sanguineus*	Thailand	This study
		Trinidad and Tobago	18
	*Hyalomma asiaticum*	China	21
		China	GenBank MH688540-41
	*Hyalomma detritum*	China	GenBank MH688542-43
	*Dermacentor marginatus*	China	GenBank MG820135-37
Wuhan tick virus 1 (WhTV1)	*Rhipicephalus* sp.	China	13
	*Dermacentor marginatus*	China	GenBank MH031780-82
	*Rhipicephalus microplus*	Thailand	This study
		China	16
		China	GenBank MH814974
	*Rhipicephalus annulatus*	China	16
		Turkey	20
Lihan tick phlebovirus (LTPV)	*Dermacentor nitens*	Colombia	GenBank MK040531
	*Rhipicephalus microplus*	Thailand	This study
		China	16
		China	GenBank MH814975-76
		Trinidad and Tobago	18
		Brazil	17
	*Hyalomma marginatum*	Turkey	19
	*Rhipicephalus sanguineus*	Turkey	20
Wuhan tick virus 2 (WhTV2)	*Rhipicephalus microplus*	Thailand	This study
		Trinidad and Tobago	18
		China	16
		Brazil	17
Changping tick virus 2 (CpTV2)	*Rhipicephalus sanguineus*	Thailand	This study
		Trinidad and Tobago	18
	*Dermacentor* sp.	China	16
	*Haemaphysalis parva*	Turkey	20
		China	16

The serological approach employed in the present study to assess the exposure of humans and mammals to viruses carried by ticks is highly dependent of the conservation of epitopes conformation. LIPS test developed by Burbelo *et al*. allows the recovery of viral antigens under native conditions using expression system in mammalian cells, limiting therefore this issue (Burbelo et al., 2010). Since it was impossible to obtain any positive control, and therefore to assess the analytical sensitivity of LIPS assay for each of the targeted antigens, we have successfully validated the protocol using the same approach targeting Hepatitis E virus (HEV), a prevalent zoonotic virus in France (Capai et al., 2019). Briefly, recombinant viral antigens expressing the external domain of the capsid of HEV were produced and further used for screening of French human sera by LIPS. The assay showed the same sensitivity as the one observed when human sera were tested in parallel by the commercial ELISA (Supplemental Table 3). In addition, LIPS sensitivity has been extensively described in different studies and demonstrated to be at least as sensitive as plaque reduction neutralization test and ELISA (Burbelo et al., 2012; Tin et al., 2017; Crim et al., 2019). We therefore conclude that the different viruses we studied are not (or very rarely) transmitted to humans and cattle (<0.017% and <0.047%, respectively). Therefore, the six selected viruses may possibly either infect other vertebrates exposed to tick bites [and especially the role of rodents and migratory birds has to be evaluated, as regards to the trophic preference of ticks (Mlera and Bloom, 2018; Tomassone et al., 2018)], or be restricted to their tick hosts.

Tick-borne flavivirus, phlebovirus, rhabdovirus, and chuviruses described in this study presented a high global prevalence (ranging from 18 to 22%), which could suggest that they are tick endosymbionts (Bouquet et al., 2017; Tokarz et al., 2018; Sameroff et al., 2019). In addition, the low degree of host restriction of these viruses, previously identified in various tick species and genera from distant geographical origins, supports this hypothesis (Table 4). Our serological results in humans and cattle also are in favor of this hypothesis. Our results show that LTPV-Thailand phlebovirus, although it has phylogenetic relatedness with Uukuniemi virus [able to infect cattle and humans (Saikku, 1973; Traavik and Mehl, 1975)], seems not be able to infect these species (or at very low prevalence). This virus, highly prevalent in *Rh. microplus* ticks, and other related bisegmented phleboviruses belonging to the same clade, should more likely be considered as tick endosymbionts that represent viral ancestors of the known trisegmented tick-borne phleboviruses, as previously suggested (Li C. X. et al., 2015). Similar conclusions could be drawn for Thai strains of tick-borne flavivirus, rhabdovirus, and chuviruses described here. However, it has been suggested that viruses presented a low degree of host restriction, and a high prevalence in ticks populations could be considered as vertebrate-borne viruses, reflecting the origin of bloodmeal of ticks (Sameroff et al., 2019). Further investigations are therefore needed to conclude on the host spectrum and get primary insights into the ecological cycle of these viruses.

The prevalence of Thailand tick thogotovirus in *Rh. microplus* ticks was inferior to 3%, suggesting that this virus possibly infects vertebrate hosts, according to Bouquet hypothesis linking prevalence of a given virus in ticks with its putative infectivity in vertebrates (Bouquet et al., 2017). Indeed, thogotoviruses are zoonotic tick-borne viruses, among which Bourbon, Dhori, and Thogoto viruses are able to infect several mammals (cattle, sheep, donkeys, camels, and buffalos) including humans, causing for the latter from febrile illness to encephalitis (McCauley et al., 2012). Apart from ticks, many arthropods were reported as alternative vectors, including mosquitoes [Batken and Sinu viruses (Frese et al., 1997; Contreras-Gutiérrez et al., 2017)] and biting midges (Temmam et al., 2016). Several evidence of a possible role of rodents as amplifying hosts or reservoirs were reported (Ejiri et al., 2018). Our survey shows that human and cattle infections were absent (or at most very rare), although this virus is phylogenetically close to known human pathogens. This finding raised the question of the mechanism of host restriction of tick-borne viruses (and more specifically of Thailand tick thogotovirus and other Oz-related tick-borne viruses) and more generally of the mechanisms that underlined the adaptation of tick-specific viruses to vertebrate-infecting tick-borne arboviruses (Li C. X. et al., 2015; Shi et al., 2015).

## Conclusions

Although identifying novel zoonotic viruses is of great importance and a prerequisite to monitor silent viral emergences, it is of equal importance to determine, among the huge number of viruses composing the virome of vectors and reservoirs, which viruses are not able to cross the species because it could lead to a better understanding of the mechanisms underlying viral emergence, and especially to understand why some viruses that are phylogenetically close to zoonotic viruses are not able to spill over. The strategy developed in this pilot study starting from the inventory of viral communities infecting a given vector by HTS to finally identify reactive antibodies possibly present in exposed vertebrate populations by luciferase immunoprecipitation is well-adapted to such considerations and could easily be transposable to other vectors/mammals interfaces.

## Data Availability Statement

The datasets generated for this study can be found in the NCBI GenBank database MN095535 to MN095546.

## Ethics Statement

The studies involving human participants were reviewed and approved by Ethical Committee of the Faculty of Tropical Medicine, Mahidol University, Thailand. The patients/participants provided their written informed consent to participate in this study. The animal study was reviewed and approved by Department of Livestock Development, Thailand.

## Author Contributions

ST, MDu, J-FC, MV-T, SMou, and ME designed and conceived the study. ST, DC, EDu, MDu, EDe, MDe, LY, and LG performed the experiments. ST and TB performed the bioinformatics analyses. MDe, SJ, AK, KC, J-FC, MV-T, and SMor realized fieldwork in Thailand. ST wrote the manuscript. ST, DC, TB, SP, MDu, SMor, SMou, MDe, and ME reviewed the manuscript.

### Conflict of Interest

The authors declare that the research was conducted in the absence of any commercial or financial relationships that could be construed as a potential conflict of interest.
