# Supplementary material for: Monitoring Silent Spillovers Before Emergence: A Pilot Study at the Tick/Human Interface in Thailand
Source: Front Microbiol. 2019 Oct 17;10:2315. doi: 10.3389/fmicb.2019.02315 (PMC6812269; doi:10.3389/fmicb.2019.02315)

Supplementary Material

**Supplemental Table 1.** Positions of the expressed extracellular regions of viral glycoproteins (GP) or nucleoproteins (NP).

| **Viral family** | **Viral target** | **Gene/Segment** | **Accession no** | **Expressed positions (nt)** |
| --- | --- | --- | --- | --- |
| *Orthomyxoviridae* | Thailand tick thogotovirus | Segment 4 (GP) | MN095542 | 35 - 1483 |
| *Phenuiviridae* | LTPV-Thailand | Segment S (NP) | MN095538 | 25 - 1455 |
| *Flaviviridae* | BLTV4-Thailand | Envelope gene (GP) | MN095535 | 64 - 540 |
| *Rhabdoviridae* | WhTV1-Thailand | Nucleoprotein gene (NP) | MN095536 | 27 - 1493 |
| *Chuviridae* | WhTV2-Thailand | Glycoprotein gene (GP) | MN095546 | 7035 - 8663 |
| *Chuviridae* | CpTV2-Thailand | Glycoprotein gene (GP) | MN095545 | 6443 - 8173 |

**Supplemental Table 2.** Sequences of forward and reverse primers used to generate the “GA-fragments”.

| **Primer** | **Sequence 5’ – 3’** |
| --- | --- |
| GA-forward | TAATACGACTCACTATAGGGCTAGCGATCGC**GCCACC***ATG*-target-specific sequence |
| GA-reverse | GTGTGAAGACGCCGCTCGAGCCGAGAGCTC-target-specific sequence |

**Supplemental Table 3.** Results of LIPS and ELISA comparative serological assays targeting HEV capsid. Fifteen human sera of French healthy donors were screened against the external capsid domain of HEV. LIPS results are expressed in relative LU (which correspond to the LU/mL measured corrected from the mean of negative controls) and ELISA results are expressed in OD (mean of the two replicates).

| Sample ID | LIPS | ELISA | Conclusions |
| --- | --- | --- | --- |
| 816-189-HOUL | 0 | 0.058 | Negative for both methods |
| 1006-B-1-110-FC | 0 | 0.047 | Negative for both methods |
| 816-43-GLAL | 0 | 0.053 | Negative for both methods |
| 816-155-ZIGU | 0 | 0.049 | Negative for both methods |
| 1006-B-1-083-LL | 0 | 0.047 | Negative for both methods |
| 1006-M-1-005-CH | 0 | 0.055 | Negative for both methods |
| 1006-B-1-104-GG | 0 | 0.064 | Negative for both methods |
| **816-66-BOLE** | **3.41E+04** | **2.168** | **Positive for both methods** |
| 1006-M-1-002-AG | 0 | 0.056 | Negative for both methods |
| 816-185-VALU | 0 | 0.048 | Negative for both methods |
| 816-44-DULA | 0 | 0.054 | Negative for both methods |
| **816-167-PIMA** | **3.05E+06** | **1.910** | **Positive for both methods** |
| 1006-B-1-109-BH | 0 | 0.050 | Negative for both methods |
| 816-214-SCAN | 0 | 0.056 | Negative for both methods |
| 816-92-KEMA | 2.84E+06 | nd | Positive for LIPS, ELISA not tested |

**Supplemental Figure 1**. Relative abundance of viral families identified in Thai ticks virome.


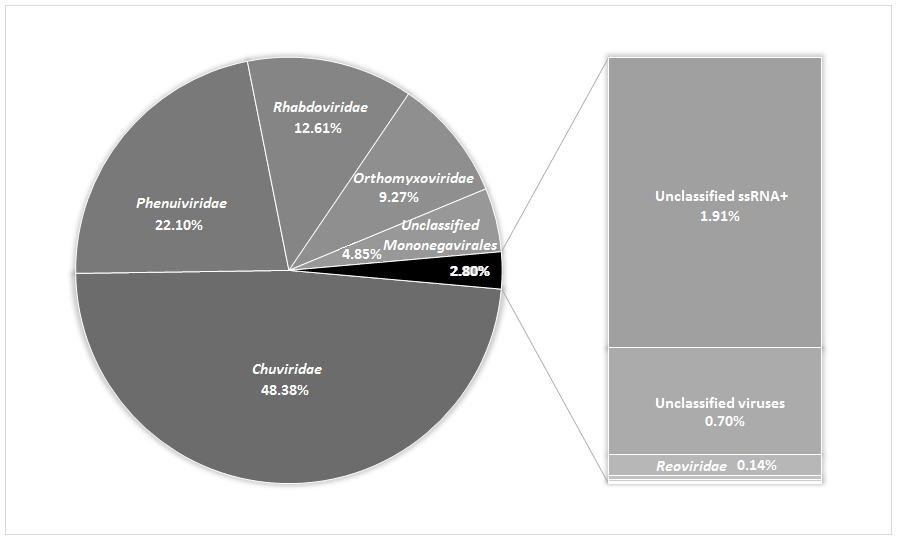

Supplement: Supplementary file 1 [file Table_1.docx]
